# Leaf Anatomical and Transcriptomic Coordination Underlies Drought Resilience in Psammophytes

**DOI:** 10.3390/ijms262110483

**Published:** 2025-10-28

**Authors:** Shangbin Shi, Wenda Huang, Yuanzhong Zhu, Hailun Yu, Cuiyun Chen, Xiaomei Peng

**Affiliations:** 1 Naiman Desertification Research Station, Northwest Institute of Eco-Environment and Resources, Chinese Academy of Sciences, Lanzhou 730000, China; shishangbin23@mails.ucas.ac.cn (S.S.);; 2 University of Chinese Academy of Sciences, Beijing 101408, China; 3 State Key Laboratory of Ecological Safety and Sustainable Development in Arid Lands, Northwest Institute of Eco-Environment and Resources, Chinese Academy of Sciences, Lanzhou 730000, China; 4 Key Laboratory of Desert and Desertification, Northwest Institute of Eco-Environment and Resources, Chinese Academy of Sciences, Lanzhou 730000, China

**Keywords:** transcriptomics, leaf anatomy, WGCNA, ecological restoration, Horqin Sandy Land, climate change

## Abstract

Global climate change-induced precipitation reduction severely threatens the sustainability of sandy grassland ecosystems. Understanding the adaptive strategies of native psammophytes is crucial for desertification control. We integrated leaf anatomy and transcriptomics (RNA-seq/WGCNA) to decipher drought resistance in three dominant psammophytes from Horqin Sandy Grassland. The finding revealed that the C3 annual/biennial herb *Artemisia scoparia* exhibited the most robust transcriptomic response, with co-expression modules linking tyrosine metabolism to cuticular thickening; the C3 semi-shrub *Lespedeza davurica* showed superior anatomical adaptation, underpinned by phenylpropanoid biosynthesis, while the C4 perennial herb *Cleistogenes squarrosa* exhibited molecular signatures of high drought sensitivity, with severe drought disrupting its flavonoid biosynthesis and circadian rhythms. In this study, the C4 herbaceous species showed stronger precipitation dependence than the C3 herbs. Our study provides molecular–anatomical insights into the ecological restoration of sandy grasslands under global change, suggesting the use of shrubs as primary stabilizers for sand fixation, alongside breeding herbaceous genotypes with optimized anatomical and transcriptomic traits to meet the needs of sustainable vegetation recovery in sandy grasslands under climate change.

## 1. Introduction

Global climate change presents significant challenges to terrestrial ecosystems, with reduced precipitation, global warming, and frequent extreme weather events making the protection and restoration of fragile ecosystems even more arduous [1]. In recent years, the northern region of China, particularly the arid and semi-arid region, has emerged as one of the areas most severely impacted by global changes [2]. Long-term meteorological data reveal that annual precipitation is diminishing, the annual average temperature is conspicuously escalating, and a trend of warming and drying is evident in the majority of semi-arid areas [3]. The temperature increase not only intensifies precipitation fluctuations [4] but also further elevates the frequency, duration, scope, and severity of extreme drought and precipitation [5,6]. These alterations in precipitation patterns have profound influences on ecosystems, agricultural production, and the social economy [7]. Previous studies have indicated that the intensification of aridity has disrupted the equilibrium of grassland ecosystems in Northern China, leading to a gradual reduction in dominant species of non-drought-resistant plants in the community [8], a decline in species diversity [9], and the prominent issue of grassland degradation [10]. Hence, strengthening research on climate change is indispensable for comprehending and predicting its impact on grassland ecosystems.

Precipitation serves as a pivotal factor shaping the structure and functioning of grassland ecosystems [11]. It is widely recognized that variations in the amount, intensity, and frequency of precipitation can modify soil moisture levels, thereby influencing the adaptability of dominant species within grassland communities [12]. Precipitation also regulates species stability and asynchrony (i.e., the tendency of species populations to respond differently to environmental fluctuations, thus stabilizing community productivity), and further affects community species richness and diversity [13]. Long-term increases or decreases in rainfall significantly enhance or reduce the productivity of grassland communities [1]. Studies have suggested that increased precipitation can offset the adverse effects of warming on grassland ecosystems [14]. Moreover, changes in precipitation patterns influence soil nutrient cycling and subsequently affect the activity of soil microorganisms and the decomposition process of organic matter [15]. Additionally, precipitation shifts during different phenological stages may potentially affect plant reproductive and dispersal capacities, subsequently altering community species composition, distribution, and richness, as well as aboveground net primary productivity [16]. Therefore, understanding the influence of precipitation on the dominant species in grassland communities will offer support for predicting the response of the grassland ecosystems to the alteration of precipitation patterns under future climate change.

Leaves are essential organs for photosynthesis and transpiration in plants and are also sensitive to external changes. Plants exposed to drought stress exhibit distinct morphological characteristics compared to other plants [17]. Drought-resistant plants exhibit specific epidermal structural features, including thickened cell walls, tight cell arrangement, and the presence of a tough cuticle and wax layer. Stomatal characteristics vary among different drought-resistant plants, but most have larger substomatal chambers to reduce transpiration [18]. Drought-resistant plants typically have smaller leaf surface areas compared to normal plants, which is achieved by reducing the surface area to minimize water evaporation or increasing leaf volume for water storage [19]. Leaf morphology also shows significant correlation with environmental adaptation strategies, including protective, conservative, tolerant, robust, and escape strategies, all aimed at reducing water loss and enhancing water storage [19]. The presence of well-developed water-storage parenchyma tissue with large vacuoles in the cells of xerophytic plants is beneficial for water retention, while sclerenchyma tissue provides mechanical support against wilting. Similar intra-specific adaptive changes in leaf morphology, such as a reduction in leaf area and closure or depression of stomata, can also be observed in response to drought stress [20].

Transcriptomics has been widely applied to study the stress response mechanisms and adaptations of plants [21,22], especially in the breeding of stress-resistant crops, such as drought-resistant soybeans (*Glycine max* (L.) Merr.) [23], rice (*Oryza sativa* L.) [24], wheat (*Triticum aestivum*) [25], etc. The drought resistance mechanism of plants in arid areas is a common research object, and 12 key genes and 5 major TFs have been identified in alfalfa (*Medicago sativa* L.) that play important roles in drought stress response [26,27]. Hormone signal transduction and phenylpropanoid biosynthesis of *Ammopiptanthus mongolicus* (Maxim. Ex Kom.) play important roles in drought response [28], and flavonoid biosynthesis genes respond to both drought and cold stress [29]. Enrichment of drought-induced significantly differentially expressed genes in *Artemisia wellbyi* for photosynthesis and transcriptional regulation functions [30]. *Astragalus membranaceus* Bge. var. *mongolicus* (Bge.) Hsiao [31], and *Panicum virgatum* L. [32], etc., have all been studied.

The Horqin Sandy Grassland, located in Northeastern China, represents a typical fragile ecosystem [33]. In recent years, regional climate change has exerted profound impacts on precipitation patterns. Studies reveal that climate change has led to a decline in annual precipitation, including an uneven seasonal distribution of rainfall, and an increase in the frequency and intensity of extreme weather events [34]. These changes have reduced water-use efficiency and exacerbated grassland degradation. Comparative research on the structural and molecular traits of psammophytes has been ongoing to uncover the adaptive mechanisms of grassland communities at the ecosystem level. Ongoing comparative studies on the structural and molecular traits of psammophytes aim to explore the adaptive mechanisms of grassland communities. In this study, we conducted a simulated precipitation reduction experiment and performed leaf transcriptome analyses on dominant plant species in the sandy grasslands. The objectives were as follows: (1) to identify key genes involved in drought stress resistance, along with their functions and gene networks; (2) elucidate the relationships between these key genes and morphological and anatomical traits; and (3) determine the metabolic pathways of the identified genes. This research provides a genetic foundation at the molecular level for the effective management and sustainable utilization of sandy grasslands.

## 2. Results

### 2.1. Analysis of Leaf Anatomical Traits

**Figure 1:**
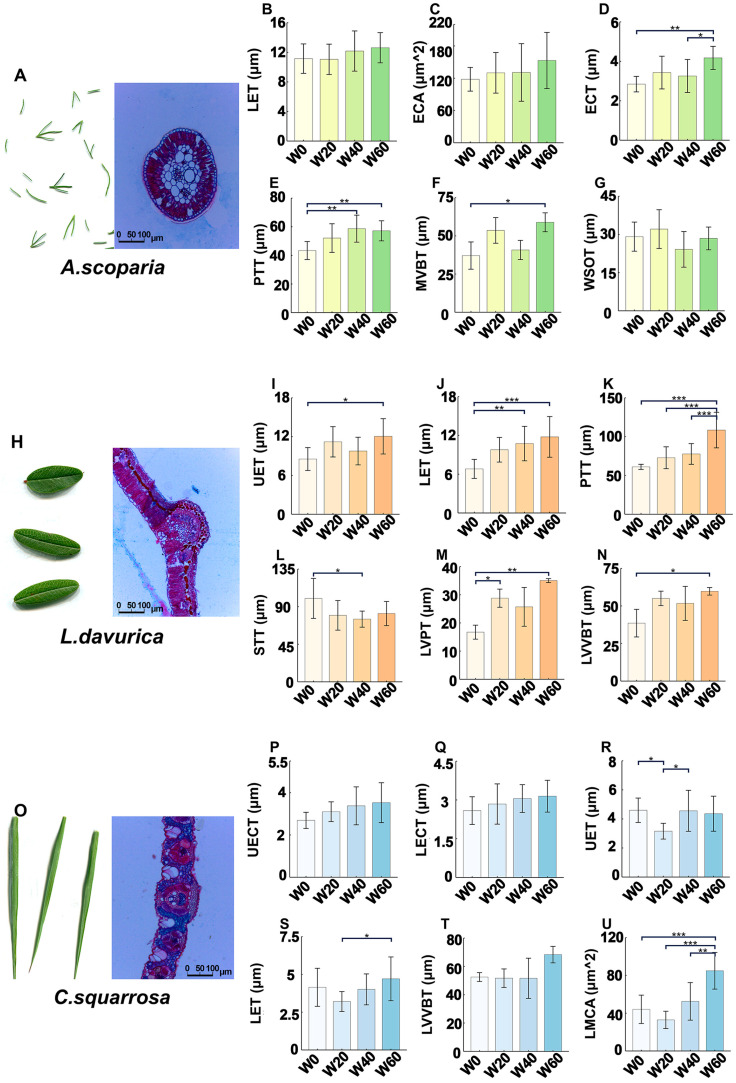
Evaluation of leaf anatomical traits in *A. scoparia* (**A**–**G**), *L. davurica* (**H**–**N**), and *C. squarrosa* (**O**–**U**). Anatomical measurement data and analysis of variance. See attached Table S1 for abbreviation notes. The x-axis represents the different treatments: W20, W40, and W60, representing a 20%, 40%, and 60% reduction in rainfall, respectively. The asterisks (*, **, ***) between columns represent significant differences at *p* < 0.05, *p* < 0.01, and *p* < 0.001.

The three species studied are typical psammophytes and representative in ecological practices of sand fixation, yet they possess distinct life forms and photosynthetic pathways. *A. scoparia* is an annual/biennial C3 herb, whereas *L. davurica* is a perennial C3 semi-shrub, and *C. squarrosa* is a perennial C4 herb. Significant and species-specific modifications in leaf anatomy were observed in response to precipitation reduction (Figure 1).

*Artemisia scoparia* exhibited the most pronounced and consistent enhancements in leaf waterproofing structures under drought stress. A marked increase in epidermal cuticle thickness (ECT) was observed, which rose significantly by approximately 15% under moderate drought (W40) and reached nearly 30% under severe drought (W60) compared to the control (W0) (Figure 1D). Concurrently, palisade tissue thickness (PTT) also showed significant, albeit smaller, increases (Figure 1E). This coordinated thickening of protective layers is a classic xeromorphic adaptation aimed at minimizing non-stomatal water loss.

*Lespedeza davurica* demonstrated a distinct anatomical strategy centered on internal tissue reorganization. The most notable change was a significant increase in the palisade-to-spongy tissue ratio. This was primarily driven by a reduction in spongy tissue thickness (STT) under moderate drought (W40), while the palisade tissue thickness (PTT) remained relatively constant (Figure 1K,L). This alteration potentially improves water-use efficiency and light capture under stress. Furthermore, traits associated with mechanical support, such as lower epidermis thickness (LET) and leaf vein vascular bundle thickness (LVVBT), showed significant increases, particularly under severe drought (W60) (Figure 1J,N).

In stark contrast, the C4 perennial herb *Cleistogenes squarrosa* displayed the weakest and least consistent anatomical response to drought. Key protective traits, such as lower epidermis thickness (LET) and lower epidermis cuticle thickness (LECT), failed to show a significant positive response under certain drought treatments (Figure 1Q,S). This lack of structural reinforcement underscores its limited capacity for anatomical adaptation to water deficit.

In summary, the three psammophytes employed divergent anatomical strategies: *A. scoparia* prioritized cuticular and epidermal reinforcement, *L. davurica* optimized internal tissue architecture and mechanical strength, while *C. squarrosa* showed minimal structural adjustment. These findings provide a clear morphological basis for the subsequent investigation of the coordinating transcriptomic mechanisms underlying these distinct drought resilience strategies.

### 2.2. Transcriptome Sequencing Annotation and Identification of Differentially Expressed Genes (DEGs)

**Figure 2:**
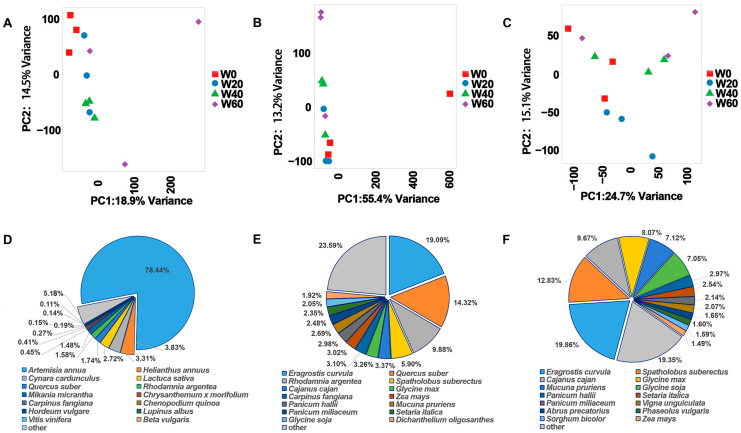
Transcriptome data analysis of three plants. Principal component analysis of gene expression in *A. scoparia* (**A**), *L. davurica* (**B**) and *C. squarrosa* (**C**). Pie charts showing the distribution and proportion of species matched with unigene BLASTx (Version 2.9.0) in the Nr protein database by *A. scoparia* (**D**), *L. davurica* (**E**) and *C. squarrosa* (**F**).

The sequencing of leaf tissues from three psammophytes, *A. scoparia*, *L. davurica*, and *C. squarrosa* yielded 604,332,366, 656,962,380, and 627,040,194 clean reads, respectively, with average Q20 and Q30 of 98.72% and 95.92%, 98.74% and 96.08%, 98.84% and 96.19%, respectively. These results indicate that the quality of our transcriptome sequencing is good. Principal component analysis (PCA) revealed that the controlled experimental conditions exerted measurable impacts on transcriptome-wide gene expression profiles in these plants, demonstrating sufficient efficacy to validate subsequent analytical deductions (Figure 2A–C). The analysis further highlighted the substantial influence of stochastic factors inherent in field conditions, providing operational guidance for frontline ecological restoration practices. Annotation results indicated that approximately 78% of the annotated sequences for *A. scoparia* were derived from *A. annua* reference sequences in the NR database. For *L. davurica* and *C. squarrosa*, the highest sequence similarity was observed with *Eragrostiscurvula*, though a significant proportion of annotations (32.1% and 41.7%, respectively) originated from unclassified or unknown functional categories (Figure 2D–F).

The sequencing analysis revealed that each of the three psammophytes had transcription factors (TFs) detected on unigenes (Supplementary Table S3), with the trend of differences was as follows: *A. scoparia* (1599) < *C. squarrosa* (1960) < *L. davurica* (2556). We discovered that the types of TFs in the three psammophytes were essentially the same, with the MYB superfamily accounting for the highest proportion. The AP2/ERF family ranked second in terms of content in both *A. scoparia* and *C. squarrosa*, and third in *L. davurica*, but the number of TFs in *L. davurica* was higher than in the other two species. The second most abundant TFs in *L. davurica* were bHLH. Additionally, the WRKY, C2C2, and NAC TF families were distributed among all three psammophytes, and their contents were relatively high. The higher number of TFs in the perennial semi-shrub *L. davurica* may reflect a requirement for a more complex transcriptional regulatory network to manage long-term growth, development, and persistent stress responses in a woody perennial life form, compared to the herbaceous species. The sequencing analysis also determined the number and length of the coding sequences (CDS). The trend of differences in the number of CDS was as follows: *C. squarrosa* (82,894) < *A. scoparia* (103,070) < *L. davurica* (156,282), and most of the lengths were between 201 and 400 bp.

**Figure 3:**
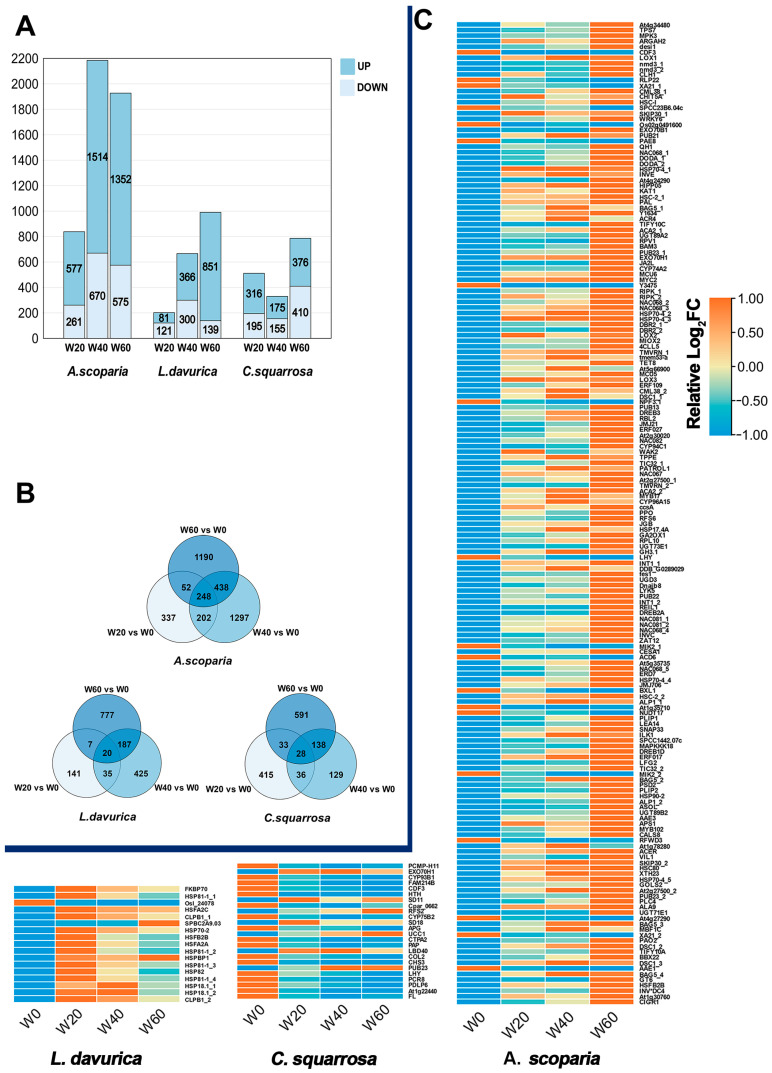
Statistical analysis of differentially expressed genes (DEGs) in three plant species under reduced precipitation conditions. (**A**) Bar charts showing a significant increase/decrease in DEGs expression levels between each experimental treatment group and the control group (W0). (**B**) The Venn diagram of DEGs, in which the positive central intersection is considered to be drought-sensitive genes (DSGs). Among the 248 DSGs in *A. scoparia*, there are a total of 177 with swissprot annotations, 20 DSGs in *L. davurica* have 17 annotations, and 27 DSGs in *C. squarrosa* have 24 annotations. The relative expression levels are shown in (**C**). Orange and blue represent relative log2FC in four conditions.

DEGs were identified using the thresholds of |log_2_fold change| > 1 and adjusted *p*-value < 0.05 (Benjamini–Hochberg correction) through comparative transcriptome analysis (Supplementary Table S2). In the case of decreased precipitation, the quantity of up-regulated genes in the three psammophytes was essentially greater than that of down-regulated genes. The number of up-regulated genes in *A. scoparia* was approximately twice that of down-regulated genes, and the number of up-regulated genes in *C. squarrosa* was approximately equal to that of down-regulated genes. Nevertheless, the number of up-regulated genes in *L. davurica* in precipitation reduction 60% (W60) was six times that of down-regulated genes. Among the three plants, the number of up-regulated and down-Regulated genes of *A. scoparia* under the precipitation reduction 40% (W40) condition was the highest, with 1514 and 670, respectively, while the number of up-regulated and down-regulated genes of *L. davurica* under the precipitation reduction 20% (W20) was the lowest, with 81 and 121, respectively (Figure 3A). When precipitation decreased by more than 40%, the number of differentially expressed genes (DEGs) in *A. scoparia* and *L. davurica* was significantly higher compared to 20% precipitation reduction, whereas the DEG quantity trend in *C. squarrosa* differed from these two species. The intersection genes of W60 and W40 in the three psammophytes were 438, 187, and 138, respectively, surpassing the values observed under the other two treatments. Furthermore, the number of shared genes of *A. scoparia* was markedly higher than that of the other two plants, reaching 248 (Figure 3B). We consider that the central intersection of the Venn diagram represents drought-sensitive genes (DSGs). Among the 248 DSGs in *A. scoparia*, there are a total of 177 with Swiss-Prot annotations, 17 with 20 DSGs in *L. davurica*, and 24 with 27 DSGs in *C. squarrosa*. Their relative expression levels are shown in Figure 3C.

**Figure 4:**
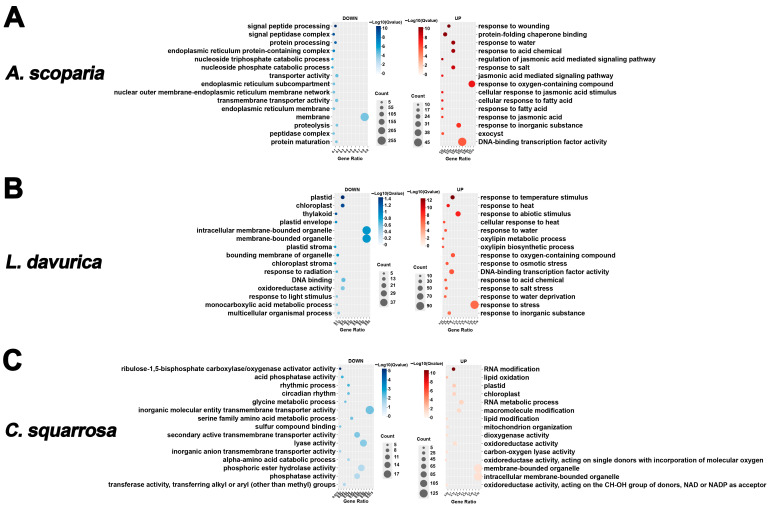
Top 15 GO terms enrichment analysis of DEGs (W60 vs. W0). GO enrichment of up-regulated and down-regulated significantly differentially expressed genes. (**A**) The top 15 GO enriched terms of DEGs in *A. scoparia* W60 compared to W0, with blue indicating downregulated genes and red indicating upregulated genes. (**B**) The top 15 GO enriched terms of DEGs in *L. davurica* W60 compared to W0. (**C**) The top 15 GO enriched terms of DEGs in *C. squarrosa* W60 compared to W0 (DEGs of W20 vs. W0 and W40 vs. W0 are shown in Supplementary Figures S1 and S2).

Enrichment analysis of the Top 15 GO terms for DEGs revealed that the “response to heat” term was significantly enriched among upregulated genes under nearly all drought treatments across the three plant species. Furthermore, *A. scoparia* exhibited enrichment for multiple stress response terms under severe drought, *L. davurica* under drought conditions of varying intensities, and *C. squarrosa* specifically under moderate drought (Figure 4A,B; Supplementary Figure S2B,C). The significant enrichment of the ‘response to heat’ term under drought is common, as both stresses share physiological challenges such as protein denaturation, triggering overlapping defense mechanisms like chaperone activity.

In *A. scoparia*, upregulated activities under mild and moderate drought were primarily enriched in protein folding. Downregulated genes were significantly enriched for terms related to DNA unwinding and transcription under mild drought (Supplementary Figure S1A), and predominantly for signal peptide processing under moderate and severe drought (Supplementary Figure S2A; Figure 4A). *L. davurica* showed similarities to *A. scoparia*, with upregulated genes under mild and moderate drought frequently enriched in protein folding (Supplementary Figures S1B and S2B). Additionally, activated stress responses were observed across all drought treatments. Downregulated genes under mild drought were mainly enriched for terms involving various enzymatic reaction activities (Supplementary Figure S1B). Under moderate and severe drought, the photosynthetic functions of plastids were significantly impaired (Supplementary Figure S2B; Figure 4B). In *C. squarrosa*, the enrichment profile of upregulated genes under severe drought shifted away from environmental stress responses and instead involved RNA metabolism and photosynthetic plastids (Figure 4C). Downregulated genes showed enrichment for extracellular structures, including cell wall-related genes under mild drought (Supplementary Figure S1C). Moderate drought enrichment centered on flavonoid biosynthesis and transcription factor activity, while severe drought enrichment involved various enzymatic reaction activities and circadian rhythms (Supplementary Figure S2C; Figure 4C).

### 2.3. Correlation Between Leaf Key Genes and Morphological and Anatomical Characteristics Traits

In this study, weighted gene co-expression network analysis (WGCNA) was employed to identify key gene modules and hub genes associated with the expression of leaf morphological and anatomical traits involved in drought adaptation. Samples with expression counts below 1 were filtered out (>90% removed), and noise reduction was performed using the Median Absolute Deviation (MAD) method, resulting in 8000 genes selected for downstream analysis. The minimum module size was set to 30, and the module dendrogram cut height was 0.25.

**Figure 5:**
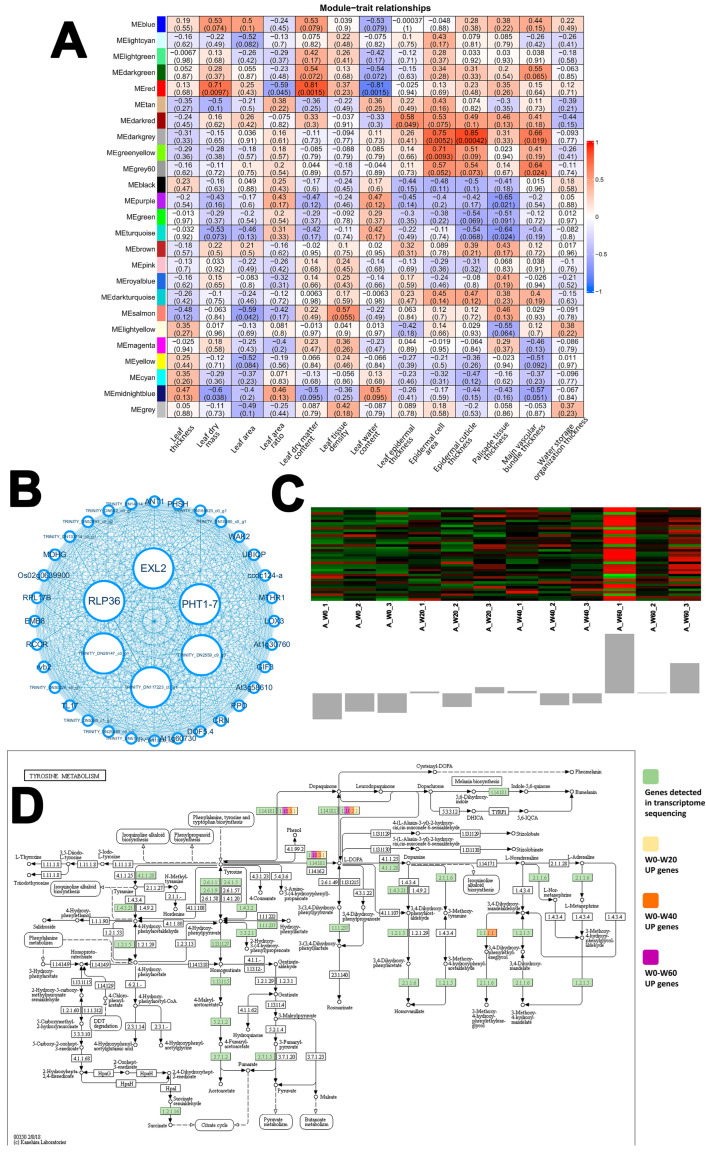
WGCNA of *A. scoparia*. (**A**) Module–trait relationships. (**B**) Dark grey module gene coexpression network. (Filter out genes with low module membership (MM) and gene significance (GS). Hub genes are represented as large circles in the middle of the network, which have the highest MM and GS. MM > 0.8, GS > 0.5). (**C**) Dark grey epidermal cuticle thickness, ME (Module Eigengene), and Gene Heatmap. (**D**) The key pathway of dark grey module gene enrichment—Tyrosine metabolism.

In *A. scoparia*, gene co-expression analysis yielded 25 distinct modules. Among these, the Dark grey module demonstrated the most compelling coordination with leaf waterproofing structures. It exhibited a strong positive correlation with epidermal cuticle thickness (r = 0.85, *p* = 0.00042), as well as significant correlations with epidermal cell area and main vascular bundle thickness (Figure 5A). This robust association suggests that the Dark grey module encompasses genes critical for constructing the leaf’s primary barrier against non-stomatal water loss. The gene co-expression network within this module, along with its hub genes (identified based on high module membership and gene significance), is presented in Figure 5B. KEGG pathway enrichment analysis of the Dark grey module pinpointed tyrosine metabolism as a central pathway. Notably, genes involved in the synthesis of dopamine and DOPA (dihydroxyphenylalanine) from tyrosine were consistently and significantly upregulated across all drought stress intensities (Figure 5D). Tyrosine-derived metabolites like dopamine have been implicated in abiotic stress responses and may contribute to cuticular fortification, providing a plausible molecular mechanism for the enhanced cuticular development observed in *A. scoparia* under drought.

**Figure 6:**
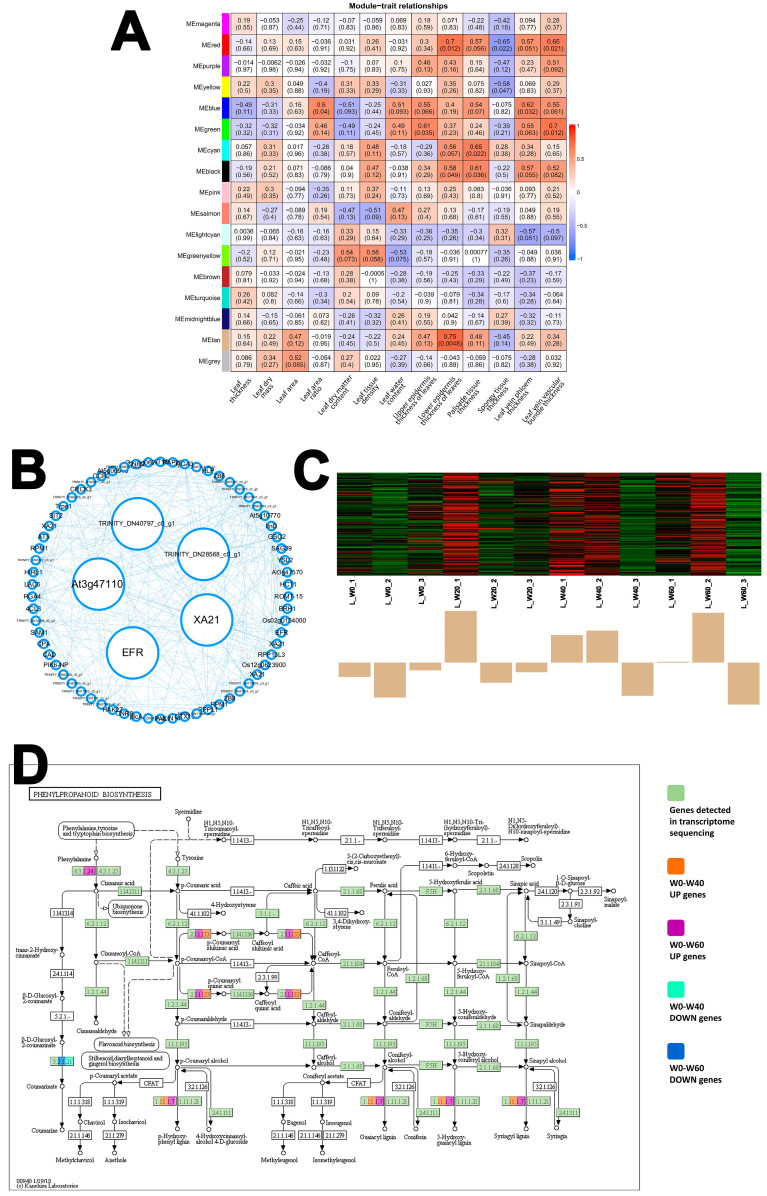
WGCNA of *L. davurica*. (**A**) Module–trait relationships. (**B**) Tan Module gene coexpression network (Filter out genes with low module membership (MM) and gene significance (GS). Hub genes are represented as large circles in the middle of the network, which have the highest MM and GS. MM > 0.8, GS > 0.5). (**C**) Tan-Lower epidermis thickness of leaves, ME (Module Eigengene), and Gene Heatmap. (**D**) The key pathway of Tan module gene enrichment—Phenylpropanoid biosynthesis.

For the semi-shrub *L. davurica*, 17 co-expression modules were identified. The Tan module emerged as the most significantly associated with leaf structural integrity, showing the highest positive correlation with lower epidermis thickness (r = 0.75, *p* = 0.0048) (Figure 6A). The network of interconnected genes within this module is visualized in Figure 6B. KEGG enrichment analysis revealed that the genes in the Tan module were predominantly enriched in the phenylpropanoid biosynthesis pathway (map00940). *L. davurica* expressed nearly all genes in this pathway (Figure 6D). Two key enzymatic activities stood out: the expression of shikimate O-hydroxycinnamoyltransferase (HCT), which catalyzes the production of caffeoyl-CoA, was significantly increased under moderate (W40) and severe (W60) drought. Concurrently, peroxidase (EC:1.11.1.7), a crucial enzyme for lignin polymerization, was also upregulated. This coordinated gene expression pattern indicates an accelerated biosynthesis of lignin, a primary component of secondary cell walls, under drought stress. This molecular response aligns with the anatomical strategy of thickening epidermal cell walls to reduce water loss and enhance mechanical strength.

**Figure 7:**
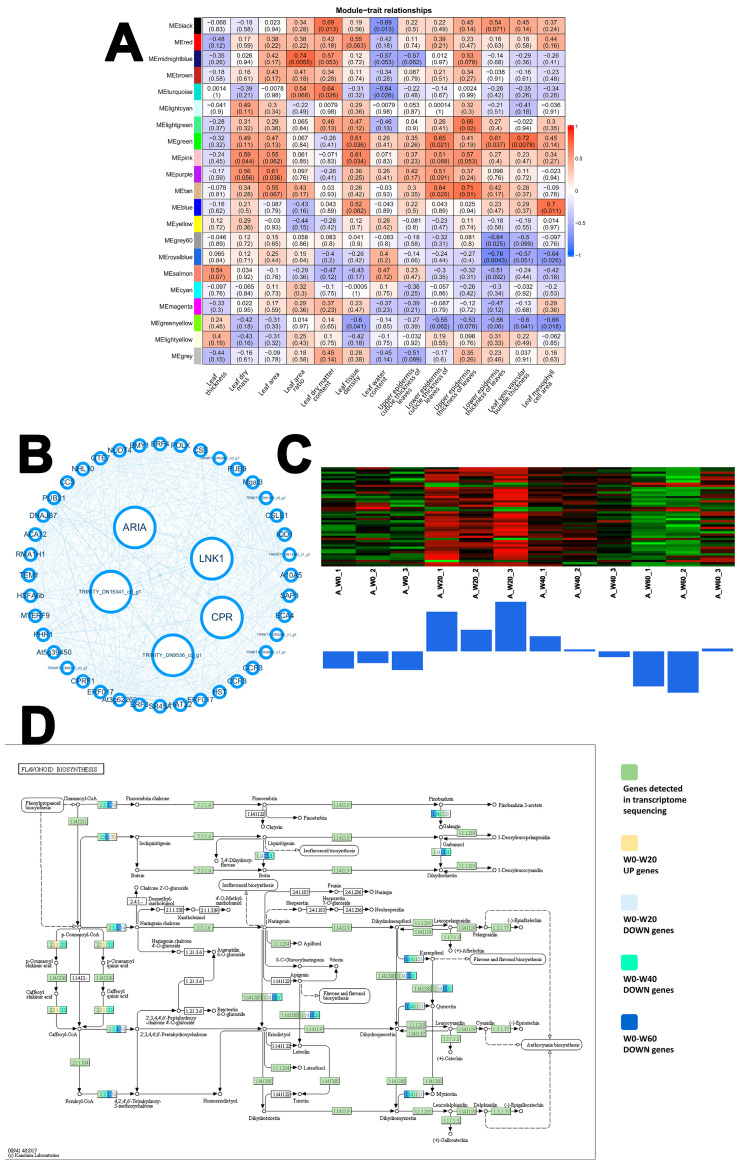
WGCNA of *C. squarrosa*. (**A**) Module–trait relationships. (**B**) Royal blue Module gene coexpression network (Filter out genes with low module membership (MM) and gene significance (GS). Hub genes are represented as large circles in the middle of the network, which have the highest MM and GS. MM > 0.8, GS > 0.5). (**C**) Royal blue—Lower epidermis thickness of leaves, ME (Module Eigengene), and Gene Heatmap. (**D**) The key pathway of royal blue module gene enrichment—Flavonoid biosynthesis.

In the C4 perennial herb *C. squarrosa*, 21 gene modules were constructed. The Royal blue module displayed a unique and telling relationship with leaf anatomy, exhibiting the strongest negative correlation with lower epidermis thickness (r = −0.76, *p* = 0.0043) (Figure 7A). This inverse relationship implies a failure or inhibition of the genetic programs required to reinforce this key protective tissue under drought. The gene network for this module is shown in Figure 7B. Pathway analysis of the Royal blue module identified flavonoid biosynthesis (map00941) as the key enriched pathway. Strikingly, critical biosynthetic genes, including flavonoid 3′-monooxygenase (CYP75B1), flavonol synthase (FLS), and chalcone synthase (CHS), were significantly downregulated across all drought treatments (Figure 7D). This widespread suppression indicates a substantial inhibition of the flavonoid biosynthesis pathway, compromising the plant’s ability to produce flavonoids like myricetin. Furthermore, the expression of HCT, also involved in this pathway, increased mildly under W20 but decreased sharply under W40, suggesting a disrupted synthesis of caffeoyl-CoA under more severe stress. The downregulation of flavonoids, which are important antioxidants, likely exacerbates oxidative damage and reflects a breakdown in the coordination between transcriptional regulation and anatomical adaptation in *C. squarrosa* under drought.

## 3. Discussion

Drought stress is one of the major challenges faced by psammophytes, and water availability is a crucial limiting factor for plant growth [35]. It affects various physiological and biochemical functions, including photosynthesis, chlorophyll synthesis, and nutrient metabolism, as well as ion absorption and transport, respiration, and carbohydrate metabolism [36]. *A. scoparia*, *L. davurica*, and *C. squarrosa* are drought-tolerant psammophytes that are widely distributed in arid and semi-arid regions. They survive in the Horqin sandy land through various physiological, biochemical, and morphological strategies. By integrating anatomical and transcriptomic data, we elucidated the distinct drought adaptation strategies of three representative psammophyte species. This comprehensive analytical framework provides novel insights into the species-specific coordination between structural modifications and transcriptional reprogramming under drought stress.

All three psammophytic plants, *A. scoparia*, *L. davurica*, and *C. squarrosa*, exhibit both significant species-specific adaptive strategies and key common response mechanisms when facing drought stress. All three plant types respond to water stress through structural adjustments (such as thickening of the epidermis/cuticle and changes in tissue ratios) and molecular regulation (differential gene expression and enriched metabolic pathways), but the specific strategies are clearly differentiated:

*A. scoparia*, as a pioneer species, exhibited the highest number of DEGs and DSGs, demonstrating the most proactive transcriptional regulatory capacity. The co-expression modules most strongly linked to its anatomical adaptations, epidermal and cuticular thickening, were enriched for cysteine–methionine [37,38,39,40] and tyrosine metabolism pathways [41,42]. It reinforces cuticular metabolic pathways to minimize non-stomatal water loss [43]. The enriched pathways in the Dark grey module are likely related to the important physiological pathways mediating the increase in cuticle layer thickness in *A. scoparia* leaves. These pathways may include the composition of cuticular waxes, such as alkanes, esters, glycols, and steroids [15], as well as isoquinoline alkaloids, Linoleic acid, and folate biosynthesis. The hub genes in the Dark grey module represent a variety of proteins in the network that regulate the physiological network of cuticle layer cells and may also affect the structural level of these cells. The potential role of dopamine metabolism [44,45,46] from tyrosine in stress response and cuticle development warrants further investigation. The enriched pathways in the Dark grey module, particularly tyrosine metabolism, are intriguing. Tyrosine serves as a precursor for various specialized metabolites, including catecholamines like dopamine. While the direct role of dopamine in cuticle formation requires further validation, it has been consistently implicated in enhancing abiotic stress tolerance and may contribute to cell wall fortification and cuticular composition [44,45,46]. Tyrosine is a precursor for a variety of specialized metabolites, including catecholamines like dopamine. Dopamine has been implicated in abiotic stress tolerance and may influence cuticular composition and cell wall strengthening. Thus, the upregulation of this pathway presents a plausible molecular mechanism underpinning the observed cuticular thickening in *A. scoparia*. This proactive transcriptional regulation likely underpins *A. scoparia*’s success as a pioneer species in arid environments.

*L. davurica* possesses superior anatomical adaptations, particularly an increased palisade-to-spongy tissue ratio [47], which is a classic adaptation for improving water-use efficiency and light capture under stress [48]. The tan module of *L. davurica* showed a strong correlation with upper epidermis thickness in the leaves. Under moderate to severe drought stress, the expression of genes related to Caffeoyl-CoA synthesis significantly increased. Thicker epidermal cell walls play a role in reducing water loss and resisting wind erosion. This suggests that while *L. davurica* is anatomically well-adapted to conserve water, but the changes in DEGs show that it suffers from metabolic impairment, particularly in photosynthesis and protein homeostasis, under prolonged drought [49].

*C. squarrosa*, being an herbaceous plant, adopts a relatively passive response strategy, activating transcriptional responses only under severe stress, indicating a lower threshold for stress perception or a more passive strategy until conditions become critical. The severe disruption of the circadian rhythm pathway is a critical finding, as the circadian clock is integral to coordinating plant responses to abiotic stress, including drought [50,51,52,53]. The circadian clock is a master regulator that coordinates physiological processes such as stomatal aperture, photosynthesis, and stress-responsive gene expression with daily environmental cycles [50,51]. Its disruption under drought likely leads to a loss of temporal coordination in these critical functions, exacerbating the cellular damage and contributing to the overall vulnerability observed in *C. squarrosa*. This transcriptional vulnerability is corroborated by its lack of positive anatomical coordination, as evidenced by the strong negative correlation between the Royal blue module and lower epidermis thickness (Figure 7A), suggesting a failure to reinforce key water-conserving structures. Although not shown in the figure, this disruption is also reflected in the KEGG enrichment of the Green and Green-yellow module. The Royal blue module of *C. squarrosa* showed a significant negative correlation with lower epidermis thickness of leaves. In the flavonoid biosynthesis pathway, the synthesis of flavonoids such as Myricetin was inhibited. The concurrent downregulation of flavonoid metabolism, often associated with oxidative stress response [54,55], points towards significant oxidative damage and a potential breakdown in regulatory coordination under severe water deficit.

*A. scoparia*, despite being an annual/biennial herb, has a drought avoidance strategy that is not dependent on perennial structures but is achieved through rapid and profound transcriptional reprogramming and cuticular reinforcement. As a pioneer species, its superior drought resilience likely stems from minimizing water loss through enhanced cuticle formation, allowing it to maintain tissue hydration and physiological activity longer than expected under drought [56]. *L. davurica*, a semi-shrub, leverages its perennial woody structure for stability and resource storage. Its superior anatomical adaptation, characterized by an increased palisade-to-spongy ratio, is a classic xeromorphic trait that enhances water-use efficiency (WUE) under water stress [57]. Our transcriptomic data confirms that this structural change is supported by the sustained activation of the phenylpropanoid biosynthesis pathway, crucial for mechanical strength. Interestingly, *C. squarrosa*, a C4 perennial herb, demonstrated the highest sensitivity to drought. This appears counterintuitive, as the C4 pathway is typically associated with higher WUE and better performance under high temperature and light stress [58]. However, C4 grasses often prioritize photosynthetic efficiency over drought avoidance. Under severe water deficit, the observed downregulation of flavonoid biosynthesis and catastrophic circadian rhythm disruption indicate a systemic failure in regulatory networks and oxidative defense, surpassing the protective capacity of its C4 biochemistry [59]. This suggests that in the context of the prolonged and severe precipitation reduction simulated in our study, the drought-avoidance mechanisms employed by the pioneer C3 species and subshrub were more effective than the physiological tolerance typically associated with C4 photosynthesis [58], which is consistent with similar findings reported in other temperate region studies [60,61].

The strategies of these three psammophytes collectively demonstrate the multi-level nature of drought adaptation: the annual/biennial herb (*A. scoparia*) relies on proactive transcriptional reprogramming to achieve structural defense; the semi-shrub (*L. davurica*) depends on anatomical optimization yet faces metabolic constraints, while the herbaceous species (*C. squarrosa*) exposes limitations in its antioxidant and circadian rhythm regulatory systems. These findings jointly reveal an intrinsic relationship between life forms and drought adaptation strategies. Our results provide a mechanistic basis for the restoration strategy of establishing a composite system with ‘woody plants as the structural framework and herbaceous plants filling complementary niches.’ The semi-shrub *L. davurica*, with its sustained investment in phenylpropanoid biosynthesis and superior anatomical adaptation (Figure 6), is well-suited to provide long-term stability. In contrast, the annual/biennial *A. scoparia* achieves short-term niche filling through rapid transcriptional reprogramming and cuticle reinforcement (Figure 5). The high drought sensitivity of the C4 perennial herb *C. squarrosa*, however, indicates that for herbaceous components, selection of genotypes with robust anatomical and transcriptomic traits, akin to *A. scoparia*, is crucial. This multi-level phased response system collectively constructs a synergistic adaptation network within plant communities in arid ecosystems. It also fundamentally challenges the traditional reliance solely on the high water-use efficiency of C4 plants, demonstrating that structural stability holds greater ecological advantage over physiological efficiency under persistent drought conditions. This suggests a shift in restoration strategies for sandy grasslands under climate change.

## 4. Materials and Methods

### 4.1. Experimental Design and Plant Materials

The study site was located at the Naiman Desertification Research Station (42°55′ N, 120°42′ E), Chinese Academy of Sciences, in the eastern part of the Horqin Sandy Land. This area features a typical sandy ecosystem with an average annual precipitation of 351.7 mm [62] and an annual average temperature ranging from 5.8 to 6.4 °C. The precipitation reduction treatments were implemented using rain-out shelters, as described in our previous study [63]. The samples were obtained from controlled natural open plots, and four drought levels were established: W0 (natural precipitation), W20 (precipitation decreased by 20%), W40 (precipitation decreased by 40%), and W60 (precipitation decreased by 60%). Each treatment level had 6 replicates, as previously reported in our earlier publication. For each treatment level, three replicates were sampled, with each sampling involving the random selection of at least 5 plants with similar growth conditions as a mixed sample. All fresh samples were stored at −80 °C for subsequent transcriptome sequencing. Leaf morphological indices were determined before the transcriptome sampling, with measurements taken from 5 randomly selected healthy plants for each replicate at each treatment level.

### 4.2. Measurement of Morphological and Anatomical Traits

Fresh samples were immediately preserved and subjected to morphological characterization. Leaf area quantification was performed using an Epson Expression series scanner Perfection V37/V370 with EPSON Perfection V37/V370 Photo Windows Driver (Ver. 3.9.2.5). Leaf thickness was measured with a digital vernier caliper (Mitutoyo, Kawasaki, Japan, ±0.01 mm resolution), with three independent replicates per sample to minimize stochastic errors. Leaf water content and leaf dry mass were determined gravimetrically by comparing fresh mass to oven-dried mass (48 h at 81 °C in a forced-air drying oven).

For anatomical analysis, fresh leaf segments were fixed in FAA solution (formalin–acetic acid–alcohol), dehydrated through an ethanol series, and embedded in paraffin [64]. Transverse sections (8–10 μm thickness) were prepared using a rotary microtome, stained with safranin-fast green, and imaged under a compound light microscope equipped with a calibrated digital camera. Epidermal, cuticular, and vascular bundle dimensions were quantified using ImageJ software (v1.53) with scale calibration against stage micrometers. The set of anatomical traits measured was tailored for each species based on its distinct morphological structures and ecological relevance, as detailed in Figure 1 and Supplementary Table S1.

### 4.3. RNA-Seq Data Processing and Transcriptome Assembly

The sample transcriptome sequencing was completed by Shanghai Majorbio Co., Ltd., (Shanghai, China). Each RNA-seq library was constructed from a pooled sample of leaf tissues collected from at least 5 randomly selected plants per replicate to account for biological variation. The quality of the raw reads was checked using FastQC. Subsequently, Trinity software (v2.11.0) was used with default parameters to perform de novo assembly of high-quality filtered reads and optimize parameters such as k-mer length, expected coverage, and minimum contig length to obtain a well-assembled transcriptome. The high-quality reads were then clustered using CD-HIT (V4.6) with a sequence identity threshold of 0.95 to remove redundancy and obtain unigenes with sequence identity.

The FPKM method was used to analyze differentially expressed genes (DEGs) in different libraries, and the edgeR package (Version 3.24.3) was utilized for DEG identification. The FPKM method was used to determine the normalized read count for each unigene and evaluate the expression level. The M-value trimming average method was used to determine the normalization factor, and the negative binomial distribution method was used to calculate *p*-values. The Benjamini–Hochberg method was employed for multiple testing adjustment. DEGs were identified based on an FDR < 0.05 and |log2FC| > 1 to determine significantly differentially expressed single genes. Unigenes with a length < 200 bp and FPKM < 1 were eliminated to avoid potential assembly errors and ensure the quality of the obtained assembly. High-quality unigenes were used for further analysis, and high-quality reads were mapped back to the assembled transcripts. Significant DEGs (*p* ≤ 0.05 and log_2_FC) were identified for further study.

A BLASTx search against the NCBI non-redundant (NR) protein database was conducted (E-value < 1.0 × 10^−5^), and Blast2GO was used for gene ontology annotation of upregulated and downregulated genes. KEGG annotation for single genes was also performed using the Blast2GO tool.

### 4.4. GO and KEGG Enrichment Analysis of DEGs

Gene Ontology (GO) enrichment and KEGG pathway enrichment analyses were performed using a hypergeometric test with a significance threshold of FDR < 0.05 in TBtools software (version v2.042) [65], and enrichment bar plots were generated.

### 4.5. WGCNA

The Weighted Gene Co-expression Network Analysis (WGCNA) package in R (v 4.4.1) (v 1.72-5) (Power selects through soft thresholding 0.8 and a minimum module size of 30) was used to construct the co-expression network, and Cytoscape (v 3.10.1) using the ‘circle’ layout, was employed to visualize the gene networks [66].

### 4.6. Statistical Analysis

Statistical analyses were performed using R software (version 4.4.1). Error bars in graphical representations denote the mean ± standard error of the mean derived from three biological replicates. One-way analysis of variance was applied to assess inter-group variability, followed by Tukey’s HSD post hoc test for pairwise comparisons. Statistical significance was annotated as follows: * *p* < 0.05, ** *p* < 0.01, and *** *p* < 0.001. All raw data underwent normality (Shapiro–Wilk test) and homogeneity of variance (Levene’s test) verification prior to parametric testing.

## 5. Conclusions

This study reveals a spectrum of drought resilience strategies: The C3 annual/biennial herb *A. scoparia* compensated for its short life cycle with a highly proactive transcriptional strategy, rapidly reinforcing its cuticle to minimize water loss; the C3 semi-shrub *L. davurica* relied on its perennial structure and sustained investment in anatomical modification and cell wall strengthening through phenylpropanoid biosynthesis; and the C4 perennial herb *C. squarrosa*, while potentially efficient in water use under mild stress, proved most vulnerable to severe drought, suffering a collapse in key metabolic and regulatory pathways. This indicates that in sandy grasslands facing increased drought frequency, drought avoidance traits may be more critical for survival than the inherent water-use efficiency of C4 photosynthesis alone. Therefore, we propose that for the ecological restoration of sandy grasslands with species analogous to those studied here, a composite system with “woody plants as the structural framework and herbaceous plants filling complementary niches” should be established. Woody species provide a stable structural skeleton, while herbaceous plants achieve short-term niche filling through rapid transcriptional responses, ultimately forming a spatiotemporally complementary drought-resistant network to enable sustainable vegetation recovery in sandy grasslands under climate change.

## Data Availability

The data presented in this study are openly available in NCBI at [https://www.ncbi.nlm.nih.gov/bioproject/], reference number [PRJNA1234718, PRJNA1235475, PRJNA1235482].

## Supplementary Materials

The following supporting information can be downloaded at https://www.mdpi.com/article/10.3390/ijms262110483/s1.
