# Supplementary material for: Leaf Anatomical and Transcriptomic Coordination Underlies Drought Resilience in Psammophytes
Source: Int J Mol Sci. 2025 Oct 28;26(21):10483. doi: 10.3390/ijms262110483 (PMC12607441; doi:10.3390/ijms262110483)
Supplement: Supplementary file 1 [file ijms-26-10483-s001.zip › Supplementary Figures.pdf]

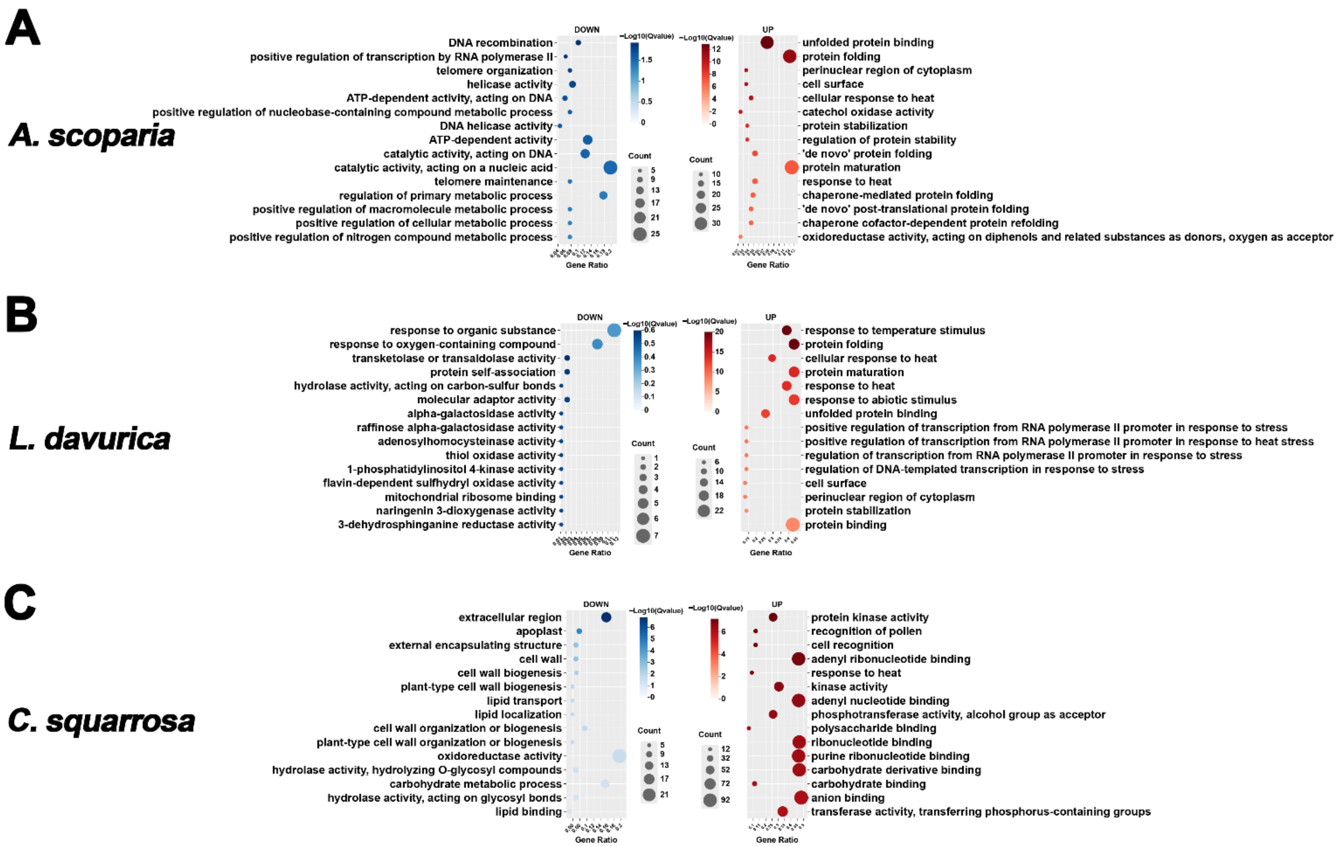

Supplementary Figure S1. Top 15 GO terms enrichment analysis of DEGs (W20 vs W0). GO enrichment of up-regulated and down-regulated significantly differentially expressed genes.

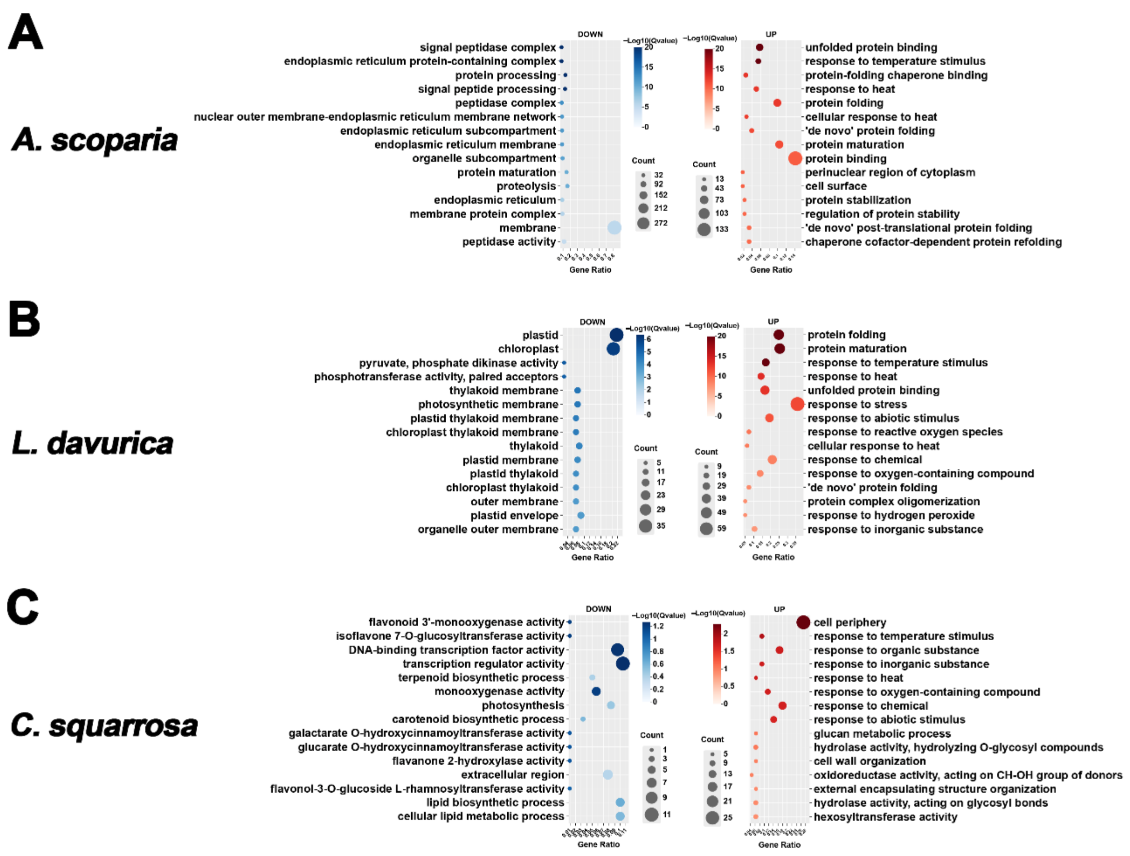

Supplementary Figure S2. Top 15 GO terms enrichment analysis of DEGs (W40 vs W0). GO enrichment of up-regulated and down-regulated significantly differentially expressed genes.
